# Advances in the Management of Localized Scleroderma: A Systematic Review of Laser Therapy and Injectable Filler Approaches

**DOI:** 10.3390/jpm14080872

**Published:** 2024-08-17

**Authors:** Fortunato Cassalia, Serena Federico, Andrea Danese, Ludovica Franceschin, Simone Amato, Francesco Gratteri, Chiara Battilotti, Francesca Caroppo, Elena Zappia, Luigi Bennardo, Anna Belloni Fortina, Steven Paul Nisticò

**Affiliations:** 1 Unit of Dermatology, Department of Medicine, University of Padua, 35121 Padua, Italy; ludovica.franceschin@studenti.unipd.it (L.F.); francesco.gratteri@studenti.unipd.it (F.G.); francesca.caroppo@unipd.it (F.C.); anna.bellonifortina@unipd.it (A.B.F.); 2 Department of Health Sciences, Magna Graecia University, 88100 Catanzaro, Italy; serena.federico@studenti.unicz.it (S.F.); elena.zappia@studenti.unicz.it (E.Z.); luigi.bennardo@studenti.unicz.it (L.B.); 3 Unit of Dermatology, Department of Integrated Medical and General Activity, University of Verona, 37100 Verona, Italy; andrea.danese_02@studenti.univr.it; 4 Dermatology Unit, Department of Clinical Internal Anesthesiologic Cardiovascular Sciences, Sapienza University of Rome, 00185 Rome, Italy; simonamato94@gmail.com (S.A.); battilotti.1938179@studenti.uniroma1.it (C.B.); nistico@unicz.it (S.P.N.); 5 Department of Medical Sciences, University of Turin, 10126 Turin, Italy; 6 Regional Center of Pediatric Dermatology and Genodermatosis, Department of Woman’s and Child’s Health, University of Padua, 35128 Padua, Italy

**Keywords:** localized scleroderma, morphea, systemic sclerosis, laser therapy, dermal filler, hyaluronic acid fillers, CO_2_ laser, dermal filler, dye laser

## Abstract

Localized scleroderma (LS), commonly known as morphea, presents a significant clinical challenge due to its chronic, inflammatory nature affecting the skin and potentially underlying tissues. This systematic review explores the innovative approach of combining laser therapy and injectable fillers, specifically hyaluronic acid, for the treatment of LS. We conducted a comprehensive literature review following PRISMA guidelines, examining articles from MEDLINE/PubMed to assess the combined efficacy of these treatments in improving both esthetic and functional outcomes for LS patients. The search yielded 64 articles, with six selected for in-depth analysis for a total of nine patients, covering a range of patient demographics and treatment types. Our review highlights cases where fractional CO_2_ laser therapy promoted long-term tissue remodeling and instances where hyaluronic acid fillers effectively addressed skin atrophy and volume loss, enhancing both immediate and long-lasting esthetic improvements. The synergy between these treatments suggests a promising dual approach, aiming to maximize esthetic outcomes and to improve the quality of life for LS patients. This review underscores the necessity of further research to establish a comprehensive, evidence-based clinical pathway integrating both treatments for managing LS, thereby enhancing patient satisfaction and addressing the multifaceted nature of this challenging dermatological condition.

## 1. Introduction

Localized scleroderma, or morphea, represents a spectrum of disorders that primarily affect the skin and subcutaneous tissues, ranging from small plaques to conditions that significantly impair function and appearance. This disease is distinguished from Systemic Sclerosis (SSc) by its predominantly cutaneous involvement and general absence of visceral complications [1,2]. The disease is divided into main subtypes including the following: circumscribed morphea, linear morphea, generalized morphea, pansclerotic morphea, and mixed subtype [3]. Mixed forms of localized scleroderma, presenting with different types of lesions in a single patient, represent a challenge to the current classification system [4,5,6,7]. Circumscribed morphea is characterized by distinct, hardened patches of skin, whereas generalized morphea causes widespread skin thickening with more relevant functional and esthetic implications. Linear scleroderma, which is more common in children, can affect deeper tissues and bones, leading to deformities. Variants such as ‘en coup de sabre’ and ‘Parry–Romberg syndrome’ affect facial appearance and may also affect joints [8,9,10,11,12,13,14,15]. The epidemiology of localized scleroderma shows a higher prevalence in women and a significant incidence in childhood [12]. Its etiology is multifactorial and involves genetic predisposition, autoimmune dysregulation, and environmental factors such as trauma and infection. Autoantibodies, including ANA and rheumatoid factor, play a key role in diagnosis and monitoring, providing insight into the autoimmune and inflammatory mechanisms of the disease [16,17,18,19]. Advances in imaging techniques, including MRI and ultrasound, have improved the assessment of tissue involvement, aiding diagnosis, and management [20,21]. With regard to therapy, in the evolving landscape of localized scleroderma management, excellent results have been reported with the use of fractional laser CO_2_ therapy and/or the strategic use of dermal fillers. The aim of this article is to conduct a literature review to evaluate cases in which fractionated CO_2_ laser therapy, dermal fillers, or a combination of both have been used to treat patients with localized scleroderma [22,23]. By examining the outcomes, efficacy, and patient satisfaction associated with these treatments, we aim to contribute to the advancement of knowledge in the management of this complex condition.

## 2. Materials and Methods

### 2.1. Study Design

We conducted a systematic review to explore the combined effectiveness of fillers and laser therapy in treating localized scleroderma (morphea). The review was conducted according to the Preferred Reporting Items for Systematic Reviews and Meta-Analyses (PRISMA) guidelines [24]. PROSPERO registration was not planned for our study.

### 2.2. Search Strategy 

We systematically searched MEDLINE/PubMed, Web of Science, and SCOPUS to detect eligible studies. The search strategy was conducted without language restrictions through February 2024. In PubMed, the following search strategy was used: laser therapy AND Morphea OR laser therapy AND localized scleroderma OR hyaluronic acid AND Morphea OR hyaluronic acid AND localized scleroderma. The search strategy was tailored to conform to the other electronic sources. The lists from each source were joined, and the duplicates were removed. Five investigators (FC, SF, AD, LF, and SA) separately evaluated the titles and abstracts of the records and removed those that fell outside the scope of the review. The full texts of all potentially eligible records were examined to dismiss those not fulfilling the inclusion criteria. Finally, the reference lists of included records were hand-searched to detect further studies of interest. Any disagreement was solved by consensus with the support of three investigators (EZ, FCaroppo, and LB). Studies not including human subjects were excluded. Studies not in English were excluded. 

### 2.3. Inclusion Criteria

All articles reporting individual cases or case series of patients with localized scleroderma in remission who underwent laser therapy, injectable filler therapy, or both were included. Articles with a suitable title, abstract, and full text were included. Non-English language articles were excluded.

### 2.4. Data Collection 

Four investigators (AD, LF, FG, and CB) independently extracted relevant data from the included articles. For each article, the study features, patient characteristics, tumor information, and outcome measures were collected. Three investigators (LB, ABF, and SPN) checked the extracted data. Any inconsistency was solved by consensus. 

### 2.5. Assessment of the Quality of Included Studies 

The quality of the included studies was assessed according to eight criteria: (i) clear criteria for patient inclusion; (ii) valid methods of disease identification; (iii) guideline-based methods applied earlier in the diagnostic and therapeutic pathway; (iv) in a number of cases, consecutive patient inclusion; (v) clear reporting of demographic data; (vi) clear reporting of clinical information; (vii) reporting of response to proposed therapy; and (viii) reporting of any adverse effects. The criteria were adapted from the Joanna Briggs Institute (JBI) Critical Appraisal Tool [25] to suit the context under investigation (evaluation of the potential use of laser therapy, injectable fillers, or both, in the treatment of localized scleroderma). Three investigators (E.Z., F.Caroppo, and L.B.) independently appraised the risk of bias of the included studies, and any inconsistency was solved by consensus with all authors. 

### 2.6. Data Synthesis 

The selection procedure was presented in a flow chart. Relevant data were extracted from the included studies and summarized in tables. The inclusion of case reports and very small case series did not allow for a meaningful meta-analysis; therefore, a narrative synthesis of the included studies was carried out.

## 3. Results 

### 3.1. Search Results and Narrative Synthesis

**Figure 1:**
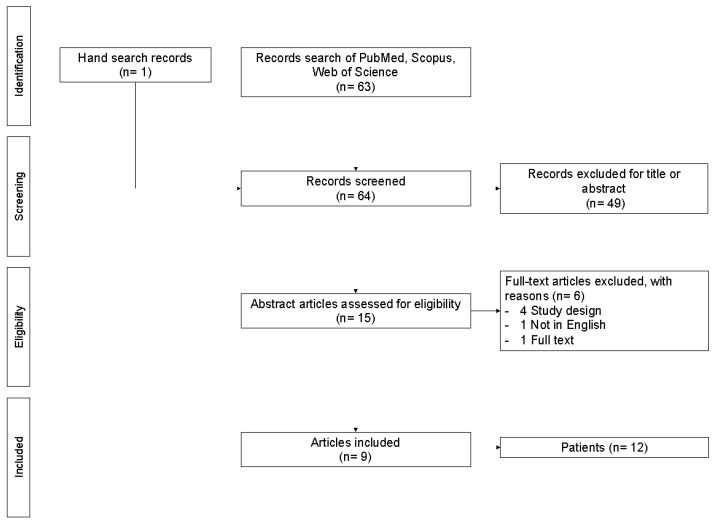
PRISMA flow chart.

The comprehensive search of key databases yielded 63 non-duplicate records. One additional eligible record was identified via hand search. We excluded 49 records according to the title or abstract. Four records were excluded for the study design, one was excluded because it was not relevant, and one record was excluded due to the language used. Finally, we identified nine eligible records for the full-text review (Figure 1). 

### 3.2. Treatment Options for Morphea

**Figure 2:**
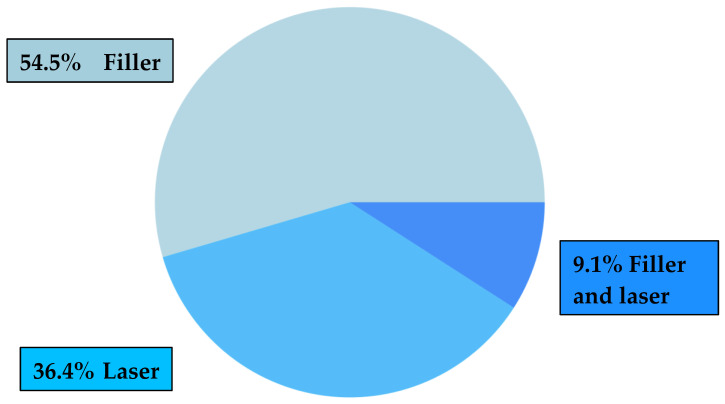
Therapy: laser, filler, or both?

Few options are available for treating localized morphea, particularly for addressing residual skin atrophy after the acute phase of the disease. Our review will focus on patients who have undergone filler treatment, laser therapy, or a combination of both treatments, which may offer potential benefits with reduced risks and recovery time. (Table 1, Figure 2). Other procedures examined in the articles included in our systematic review, which demonstrated significant clinical benefits, involved surgical interventions such as total resection of atrophic lesions, the use of synthetic tissue grafts and matrices, or autologous tissue grafts like autologous fat. However, these procedures carry considerable clinical risks and require lengthy recovery periods for patients.

### 3.3. Hyaluronic Acid Filler and UVA1 Phototherapy

In the case report by Choksi et al., excellent results were achieved in terms of skin texture and softening of the scar with the UVA1 phototherapy treatment associated with injections of hyaluronic acid fillers [26]. Hyaluronic acid injections have proved to be an excellent alternative to other, more invasive treatments. However, it should be borne in mind that in the results of these studies, the corrective effects of the treatments used may be reversed during flare-up of the disease. Autoimmune diseases are subject to fluctuations in their course; therefore, the course of autoimmune disease is often relapsing–remitting. The natural evolution of morphea is spontaneous regression that often results in a permanent atrophic scar. 

### 3.4. 585 nm Pulsed Dye Laser

Another treatment option, considered in the case report by Daniel Eisen et al. [27], is the 585 nm pulsed dye laser, which is typically used in the treatment of hypertrophic scars and keloids. In this case, four treatments were performed at bimonthly intervals. The improvements obtained were documented by skin biopsies that showed significant changes in histology. The clinical effect was positive after six months of treatment, with restitutio ad integrum of the hypertrophic scars, although the exact mechanism has not been clarified. More controlled studies are therefore needed to determine the role of the 585 nm dye laser in the treatment of this condition. 

### 3.5. Microcannula Filler Injections

Another very interesting treatment option was the use of microcannula with blunt tips for injections of soft tissue fillers. The advantage is that large areas with few entry points can be treated. In the case report included in this review, the hyaluronic acid filler was injected into a girl’s forehead by means of a microcannula inserted in the periosteal plane with the retrograde linear technique. The clinical benefit with this technique was obtained by minimizing the risks. It is desirable to create guidelines to standardize the microcannula injection technique. 

### 3.6. Hyaluronic Acid Filler: Case Series 

Among the studies, we have included a case series by Agnieszka Owczarczyk-Saczonek et al. which shows how hyaluronic acid filler injections are among the safest and most effective techniques [22]. Hyaluronic acid filler injections improve atrophy and skin texture and stimulate the production of new connective tissue, also promoting a long-term effect. To achieve excellent results, it is necessary to choose high-quality fillers that meet the approval standards of the EMA and FDA [27]. It is necessary that the technique is performed by an experienced professional. To increase the effectiveness and maintenance of the results obtained, the use of a fractional ablative CO_2_ laser can be considered. However, experimental studies are needed to explore the efficacy and safety of such treatments and to standardize appropriate treatment protocols. 

### 3.7. Hyaluronic Acid Filler: Clinical Effects

In the study by Thareja et al., the positive clinical effects of hyaluronic acid fillers are demonstrated [28]. Compared to the others, this case report highlights a fundamental aspect that, in some areas of ‘en coup de sabre’ lesions, the skin can be somehow linked to the underlying structures. For those areas of atrophy related to areas further down on the face and less towards the scalp, the hyaluronic acid filler will not be enough to correct the defect, and more invasive procedures may be needed. In the patient of the clinical case considered, in fact, the clinical improvement was noticed above all in the central part of the face for such reasons. The cosmetic result can improve evenly with repeated interventions at regular intervals for a long time. 

### 3.8. Combination Therapy: Systemic Methotrexate and Excimer Laser

The patient of the case report by Hanson et al. received a histological diagnosis by means of a morphea biopsy. The patient had previously been treated with intralesional steroids, calcipotriene ointment, and methotrexate. After four months of treatment with methotrexate, the plaque experienced a slight clinical improvement, and after six months, excimer laser treatment was combined twice a week for seven months. The patient had received a cumulative dose of 400 mg methotrexate in 19 months and had undergone a total of 34 excimer laser treatments for a maximum of 2200 mj/treatment. 

Several studies have shown significant clinical effects after an average of seven treatments. The exact cellular mode of action of the excimer laser is not known, but it appears to work by inhibiting T cell proliferation, altering the mechanisms of apoptosis, and decreasing the production of interleukins 7 and 8. However, further studies are needed to assess the synergistic effect of methotrexate and excimer lasers in the treatment of localized morphea [29]. 

### 3.9. Morphea Treatment for Functional Recovery: Fractional Ablative Laser

The case report by Kineston et al. investigates the treatment of ankle contracture related to localized morphea [30]. 

Although the long-term effects of fractional ablative laser treatment in contracture due to atrophic morphea scars are not known, the gradual remodeling of the scar over a period of months, with a cumulative effect, was found. The procedure consisted of a single pass and single, no overlap, 50 mj pulse. However, unlike traumatic scars, atrophic scars due to localized morphea can undergo reactivation with reactivation of the disease. The patient achieved a range of motion recovery with scar softening. The patient stopped UVA-1 therapy and continued concomitant treatment with methotrexate and topical agents. The benefits were maintained over 4 months of treatment. 

### 3.10. Polymethylmethacrylate Fillers

In the study by Franco JP et al., the authors choose polymethylmethacrylate (PMMA) as the filler material for the treatment of residual atrophy. This material is a permanent, biocompatible, non-toxic, non-mutagenic, and immunologically inert filler. The injection of PMMA causes a reaction like that generated with foreign bodies, as it induces the proliferation of giant cells of the immune system around PMMA particles. PMMA already finds application in various types of lipodystrophies with different etiologies. PMMA represents a valid therapeutic option for the esthetic improvement of residual scars after scleroderma and represents a valid alternative to more invasive surgical procedures [31]. 

### 3.11. Differential Diagnosis and PDL Laser

Another aspect to consider is the differential diagnosis between vascular malformations such as port-wine stain and erythematous pattern morphea. In the study by Ng SS and Tay YK et al., after the treatment of residual atrophy of the patient with the PDL laser, there was an increase in fibrosis, which clarified the diagnosis of morphea, as the skin biopsy had not clearly revealed a histological diagnosis of morphea [32]. In doubtful cases, it is better to delay treatment with PDL as it could trigger an increase in fibrosis, even if this has not yet been proven. A study by Kakimoto et al., excluded from our review because it did not meet the criteria of inclusion, shows that the intense erythema in the initial stages of a linear morphea can mimic a vascular malformation. However, the side effects correlated to the treatment with PDL, such as vesiculation and hypopigmentation, can help to identify this as not vascular malformation [33].

**Table 1:** Eligible records.

Authors	Year	N	Sex	Age	Anatomical Site	Laser Therapy	Filler
Owczarczyk-Saczonek et al. [22]	2020	3	F	17	face	no	yes
			F	16	face and trunk	no	yes
			F	70	face and trunk	Fractional ablative CO_2_	yes
Choksi AN & Orringer JS [26]	2011	1	M	20	face	no	yes
Eisen D et al. [27]	2002	1	F	14	face	Pulsed dye laser	no
Thareja et al. [28]	2015	1	F	44	face	no	yes
Hanson A et al. [29]	2014	1	F	17	trunk	Excimer laser	no
Kineston D et al. [30]	2011	1	F	27	foot	Fractional ablative CO_2_	no
Franco JP et al. [31]	2016	1	M	14	face	no	yes
Ng SS & Tay YK [32]	2015	1	F	7	face	Pulsed dye laser	no
Sivek R & Emer J [34]	2014	1	F	N/A	face	no	yes

## 4. Discussion

### 4.1. Dermal Fillers

The use of dermal fillers, notably hyaluronic acid, has become a cornerstone in the realm of medical procedures, establishing a track record of safety and efficacy across a broad spectrum of applications [30]. Although popular perception often relegates hyaluronic acid fillers to the realm of esthetic enhancement, it may be useful to explore their potential therapeutic role beyond mere cosmetic improvement. Particularly in the context of challenging dermatological conditions such as morphea, hyaluronic acid fillers emerge as a remarkably beneficial intervention, offering substantive relief and improvement to patients afflicted by this disabling condition [34,35,36]. Hyaluronic acid, a molecule naturally endemic to the dermal layers of the skin, boasts properties that are fundamentally rejuvenating and restorative. Its intrinsic ability to hydrate and volumize the skin is pivotal, especially in areas compromised by atrophy or substantive loss of tissue [36]. This action not only provides immediate visual and structural enhancement but also engages the skin’s biological processes to stimulate the production of endogenous collagen [31]. This dual action—both immediate and progressive—underscores the therapeutic value of hyaluronic acid fillers, offering not just a temporary cosmetic fix but potentially ushering in long-term dermatological benefits. The specific case of ‘en coup de sabre’ lesions, characterized by pronounced skin atrophy and volume loss, exemplifies the therapeutic efficacy of hyaluronic acid fillers. The rationale for selecting hyaluronic acid fillers for this purpose is deeply rooted in the pathological impact of the disease, which tends to induce profound alterations in skin tissue. By restoring volume, these fillers can significantly ameliorate the original contours of the affected areas, thereby enhancing symmetry and mitigating the visible impact of the lesions. However, it is crucial to temper expectations with a realistic understanding of the nature of this treatment. While hyaluronic acid fillers offer a valuable avenue for managing, for example, the skin manifestations of ‘en coup de sabre’ lesions, they do not constitute a permanent cure [37,38]. The biodegradable nature of hyaluronic acid necessitates periodic reapplication of the filler to sustain the esthetic results achieved. This characteristic underlines the importance of ongoing management and patient commitment to maintaining the therapeutic benefits over time.

### 4.2. Laser Therapy

CO_2_ laser therapy has emerged as a promising approach to the treatment of localized scleroderma, offering both esthetic and functional benefits. This technology works by creating microscopic channels in the skin that target the sclerotic bands characteristic of morphea. Patients experience immediate improvements in function and esthetic appearance through the reduction in tension and contractures. In addition, the fractional CO_2_ laser facilitates a controlled healing response, promoting organized collagen formation and long-term tissue remodeling. This innovative approach not only provides immediate relief, but also triggers a cascade of healing processes, promising sustained improvements beyond the initial treatment time [22,35,39,40,41]. In recent studies, various laser treatments have shown potential in the treatment of localized scleroderma. Tawfik et al. (2013) investigated the immunohistochemical effects of pulsed dye laser therapy; their results showed a reduction in skin induration and favorable changes in cellular markers, in particular an increase in CD34+ cells and a decrease in factor XIIIa+ cells. These changes suggest improved extracellular matrix remodeling and reduced fibrosis, highlighting the therapeutic benefits of pulsed dye laser therapy [42]. In addition, Guo et al. (2023) investigated the use of fractional erbium: yttrium aluminum garnet (Er: YAG) lasers in a mouse model of morphea. The results showed that fractional Er: YAG treatment led to a reduction in disease severity, characterized by the thinning of the dermal layer and a reduction in fibrosis, as indicated by lower levels of the markers MMP1 and TGF-β1. These results support the efficacy of fractional Er: YAG lasers in improving tissue quality and reducing fibrosis, providing encouraging evidence for the use of different laser technologies in the treatment of localized scleroderma [43].

### 4.3. Synergy between Fillers and Laser Therapy: ‘Two Is Better’?

In the dynamic field of dermatological treatments, the exploration of synergistic methodologies presents an innovative approach to managing complex conditions such as localized scleroderma. This article delves into the potential of combining filler injections and laser therapy, a dual strategy aimed at enhancing both the functional and esthetic outcomes for patients suffering from this challenging disorder. Fillers, particularly those based on hyaluronic acid, provide swift and noticeable enhancements in the appearance of scleroderma lesions by filling in depressions and correcting contour irregularities. This immediate cosmetic correction is crucial for patients seeking quick relief from the visible manifestations of the disease [35,36,37,38]. On the other hand, laser therapy, especially fractional CO_2_ laser treatment, goes beyond surface-level improvements to effectuate substantial changes within the skin’s architecture. By promoting collagen remodeling and enhancing skin elasticity, laser therapy contributes to long-lasting improvements in skin quality, which are essential for addressing the deeper structural issues associated with morphea [40,41,42]. The rationale behind this integrative approach is not merely to amplify the esthetic benefits, but also to tackle the underlying pathological alterations characteristic of localized scleroderma. This strategy may offer a holistic solution that has the potential to slow disease progression and reduce the risk of relapse by addressing surface irregularity with fillers, while promoting healing and the remodeling of deeper tissue with laser therapy. The aim is to not only extend the duration of clinical improvement but also to confront the cyclical nature of morphea, offering patients a more stable and long-term resolution of their condition. Moreover, this synergistic treatment model underscores the importance of a tailored therapeutic approach. By carefully planning the application of fillers and scheduling laser therapy sessions to complement each other, dermatologists can create a personalized treatment regimen that addresses both the immediate cosmetic concerns, and the longer-term structural challenges posed by morphea. This customized strategy enhances patient satisfaction by providing a comprehensive response to the various aspects of the disease, thereby potentially diminishing the psychosocial burden and improving the overall quality of life for those affected [44,45].

## 5. Conclusions

This review underscores the significant potential of laser therapy and injectable fillers in enhancing both esthetic and functional outcomes for patients with localized scleroderma in remission. Treatments such as hyaluronic acid-based fillers and CO_2_ laser therapy have demonstrated considerable promise in improving skin texture, elasticity, volume loss, and contour irregularities. The findings suggest that these treatments can work synergistically, providing both immediate and long-term benefits. This combined approach could lead to greater patient satisfaction and an improved quality of life by addressing both cosmetic and functional concerns. However, the current evidence is constrained by several factors. Most of the studies reviewed are small, non-standardized case series, which include diverse patient demographics, treatment protocols, and outcome measures. These limitations make it challenging to draw definitive conclusions about the efficacy and safety of these treatments. To build on these preliminary findings, future research should focus on standardizing treatment protocols to ensure consistency and comparability across studies. It is also essential to document follow-up times to assess the durability of treatment effects and to monitor side effects and remission periods to better understand the long-term safety and efficacy of these treatments. In conclusion, while the combination of laser therapy and injectable fillers appears promising for improving the quality of life in patients with localized scleroderma in remission, further research is needed. By addressing the current limitations and focusing on these key areas, future studies can provide more robust conclusions and help develop tailored therapies that significantly enhance the quality of life for individuals with this challenging condition [44,45,46].

## Data Availability

The data presented in this study are available on request from the corresponding author.
